# Material precarity and well-being among LGBTQ+ older adults in South Texas: findings from a community-engaged needs assessment

**DOI:** 10.3389/fpubh.2026.1941276

**Published:** 2026-09-29

**Authors:** Ian Johnson, Rogie Royce Carandang, Raeann Cruz, Vincent Luong, Eva Rivera

**Affiliations:** 1 Boston University School of Social Work, Boston, MA, United States; 2 School of Public Health, University of Texas at San Antonio, San Antonio, TX, United States; 3 UT San Antonio College for Health, Community, & Policy, San Antonio, TX, United States; 4 University of Washington School of Social Work, Seattle, WA, United States

**Keywords:** housing precarity, hunger, Latino/Hispanic, LGBT, material insecurity, older adult

## Abstract

**Background:**

LGBTQ+ older adults experience compounded vulnerabilities later in life, but most knowledge production about LGBTQ+ older adult experiences have occurred in coastal urban communities. This mixed-method community-engaged study aimed to (1) describe the occurrence and forms of material precarity among a sample of older LGBTQ+ adults in South Texas, (2) examine interactions between material precarity, health and social experiences; and (3) develop a cursory understanding of a community-driven LGBTQ+ aging agenda in the South Texas region.

**Methods:**

The research team deployed descriptive statistics, chi-square and Fisher’s exact testing using community survey data (*n* = 93), as well as a thematic analysis of interviews (*n* = 13) and town halls (*n* = 2) guided by interpretive description.

**Results:**

The survey indicated high rates of material precarity across four domains: housing, food, utilities, and prescription coverage. Material precarity in the survey sample was not significantly linked to sexual orientation, gender identity, or combined SOGI identity. Precarity was socially patterned, with household size, loneliness, social support, and chronic disease burden emerging as statistically significant correlations with various domains, respectively. Qualitative findings added depth to survey findings and identified three needs: (1) LGBTQ+ affirming affordable senior housing; (2) health justice efforts for LGBTQ+ older adults; and (3) social programming to address loneliness, age segregation, and loss of role in community. Findings highlight distinct vulnerabilities faced by LGBTQ+ older adults across the South Texas region.

## 1 Introduction

### 1.1 Background

Despite cultural narratives about successful aging and retirement, many older adults are facing growing material precarity—a multidimensional uncertainty about one’s financial wellbeing shaped by economic and social forces (1). Economic inequality is pronounced among generational cohorts entering older adulthood (57), a trend that has worsened since the COVID-19 pandemic began (58). Lifelong financial insecurity is correlated with numerous negative health and mental health outcomes in later life (59). For example, persistent financial strain predicts poorer self-rated health, greater functional limitation, and elevated depressive symptoms even after accounting for economic circumstances, consistent with allostatic load models of cumulative physiological wear (60). Among older Mexican-origin adults in South Texas specifically, sustained financial strain has been linked to depressive symptoms and loss of daily functioning (2), and housing insecurity compounds these effects through longitudinal psychosocial and biological pathways (3).

LGBTQ+ adults in the U.S. face higher material hardship than cisgender and heterosexual peers (4). Codified exclusion from critical safety-net resources, such as food and nutrition assistance (61), emergency shelters (62), housing assistance (63), utility assistance (64), and aging supports (65), serves to maintain disparities among LGBTQ+ people. Macroeconomic shifts toward precarious work contribute to economic instability among LGBTQ+ people, especially those navigating multiple intersecting oppressions (66). Systemic material hardship has a cyclical relationship to psychological distress and health inequities (5). Transgender older adults and LGBTQ+ older people living with HIV (67) have noted isolation (6)and vulnerability within healthcare and aging care systems, limiting how they choose to access and utilize services. Simultaneously, LGBTQ+ older adults also demonstrate internal resilience and collective community power in the face of oppressive force (5, 6), which can drive positive material and health outcomes.

Of the 7.23 million Texans aged 55 and older (68), it is estimated that nearly 400,000 are LGBTQ+ (7). Existing U.S.-based research rarely examines experiences of older LGBTQ+ adults beyond urban coastal settings (69). The geopolitical and sociocultural contours of South Texas shape how aging, material precarity, culture and ethnicity, and gender and sexual orientation intersect. Texas’ legislative and sociopolitical climate offers less protections than other parts of the country, which increases LGBTQ older Texans’ threats to health, mental health, and material security (8). For example, State Bill 1257 disincentivized insurance companies from covering gender-affirming care, and all state-funded healthcare organizations were banned from offering trainings on health equity for people with minoritized sexualities or gender expressions (9). The health disparities associated with material-need insecurities are higher among LGBTQ+ people living in the U.S. South compared to other regions (70, 71). A statewide community survey published by Texas Pride Impact Funds (10) indicated urgent needs for LGBTQ older adults, noting that rates of poor mental health were higher after age 55 than earlier stages of adulthood and insufficient financial resources were indicated as a major source of stress.

Texas has been a top destination for domestic migration in recent years, driving housing and cost of living prices and thus worsening material inequity (72). Many interventions aimed at addressing material hardship are supported by municipal and state governments (73), rendering geographically specific research on LGBTQ+ older adults necessary and urgent. At the same time, there has been a strong call to action to make Age-Friendly Cities and Communities efforts LGBTQ+ inclusive (11). South Texas has been the primary research site on the Latino Health Paradox (12, 13, 74), which has sought to understand the complex relationship between extended mortality and migration from Mexico and other parts of Central America. Given that LGBTQ+ research has not adequately collected representative data from Latine sources (75), the demographics and cultural orientation of the study setting offer the potential for insight into culturally bound needs and assets among LGBTQ+ older people.

### 1.2 Study aims

This community-engaged needs assessment started through a partnership with a community LGBTQ+ organization to develop a baseline, exploratory understanding of material precarity and related psychosocial factors among older LGBTQ+ South Texans. The study aimed to describe the occurrence and forms of material precarity among a non-representative sample of older LGBTQ+ adults in South Texas and to examine how housing, health, loneliness, and social support shape regional experiences of insecurity across the life course.

## 2 Methods and materials

### 2.1 Study design

This is a pragmatic study (14) that emerged from ongoing engagement with a local LGBTQ+ service organization, initially as an MSW practicum site. Interns and service professionals anecdotally observed a need for a research partnership to assess the needs of older adults, given a gap in regional services and increased community demand. The primary investigator received internal grant funding and completed institutional IRB for the project in March 2025. Figure 1 outlines the study procedures.

**Figure 1:**
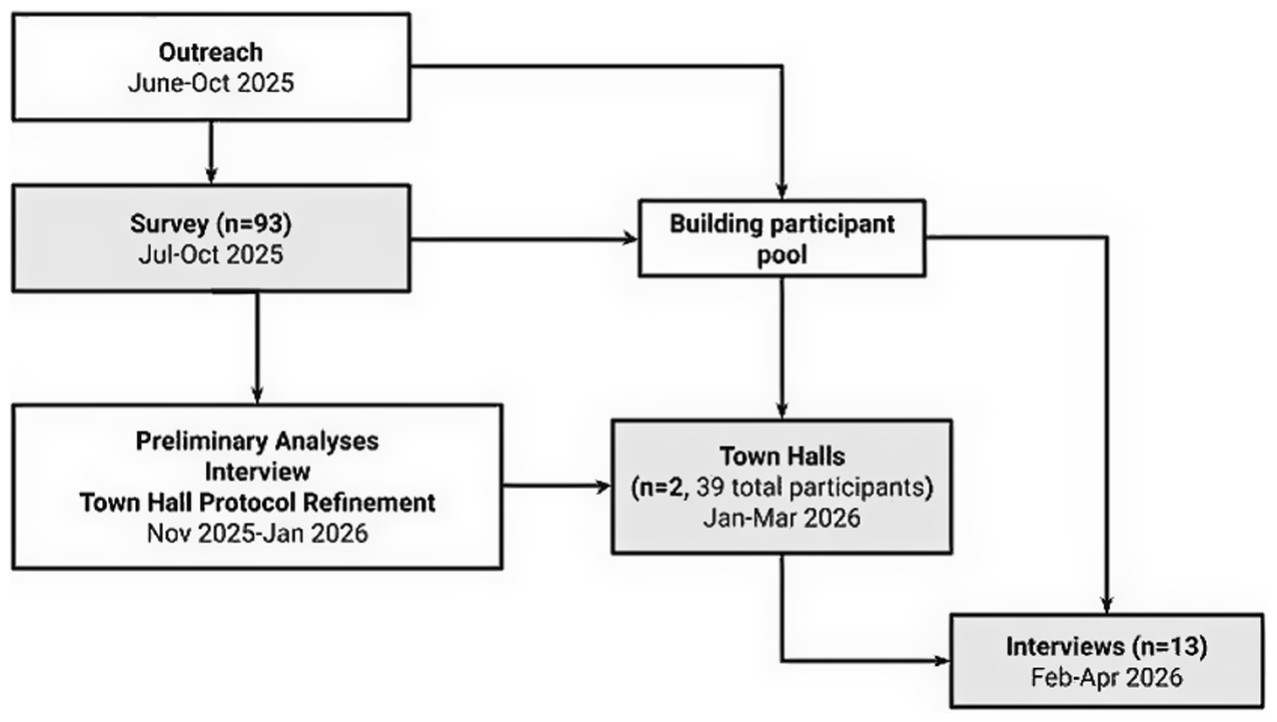
Study procedures.

### 2.2 Collection procedure

The research team circulated flyers and paper surveys with regional LGBTQ+ services, sexual health services, LGBTQ+ owned businesses, LGBTQ+ affirming faith organizations, social groups, and advocacy groups, Area Agencies on Aging, city senior services, and non-profits connecting communities with basic needs. Electronic flyers were also posted on social media (e.g., Facebook, Instagram) through geographic and identity-based groups. Surveys were open from July 1 to October 31, 2025 due to recruitment challenges and geographic spread. Surveys were anonymous. Upon completion, respondents were directed to a page with the email and telephone contact information of the primary investigator if interested in participating in town halls and interviews. Recruitment materials and surveys were available in both English and Spanish.

In January and February 2026, the first and third author hosted open forum 90-min town halls in San Antonio and the Rio Grande Valley. Town hall participants were provided with a verbal description of the informed consent process, and no identifying information was collected. Town halls followed a rapid prototyping protocol. Rapid prototyping involves deploying a series of questions and activities for collaboratively developing solutions to an emergent or understudied problem (76). In town halls, the research team presented preliminary survey results and used a protocol to elicit conversation. The protocol included probing the audience about perceived accuracy of data, emotional reactions and responses, personal alignment with data, community priorities considering survey findings, and curiosities for future qualitative data collection. Town halls were also used as recruitment forums for interviews. In March and April, the first author conducted semi-structured interviews in-person, over the telephone, and virtually. Potentially identifiable information was redacted from transcripts by the first author prior to team analyses. Following guidelines for multilevel mixed method analysis (77), the descriptive and inferential statistical findings from the surveys and rapid analysis of short-form qualitative survey data informed interview and town hall protocols. Interviews used the same eligibility criteria as the survey and followed a semi-structured protocol that included open-ended questions about personal histories (e.g., relationship to South Texas, connection to the LGBTQ+ community, lifetime experiences with precarity), present realities (e.g., current programming/service needs), and recommendations for the future.

### 2.3 Data collection instruments

The survey instrument was 34 items and consisted of seven sections: housing, food, utilities, technology, health, social life, and demographics. The development of the survey instrument was guided by an existing operationalization of multiple forms of material need insecurity (15). Specific standardized questions were chosen to have future capacity to conduct comparative analyses with local and federal datasets, including housing type, household members, home ownership, household activities, medical care activities, food insecurity, race, ethnicity, and language, veteran status, and insurance plan in the National Health and Aging Trends Study questionnaire (16); self-rated health (78), household energy insecurity and coping strategies (79), and cost-related medication non-adherence (17). Other questions replicated those that had recently been developed for community partner reports (80), including options for sexual orientation and gender identity, chronic health conditions, and the addition of two questions about cost-related medication non-adherence related to home remedies and over the counter drug substitutions. The team chose to measure social support through two pre-validated measures. The Social Support Scale measures actionable support across four domains: attention, appreciation, assistance, and advice. Respondents could choose the extent to which each statement felt true on a scale of 1(strongly disagree) to 5 (strongly agree). The UCLA Loneliness Scale (18) measures perceptions of subjective social connections, which is widely recognized and used in approximately 27% of loneliness studies. The scale measures loneliness across three domains: companionship, social connectedness, and perceived isolation from 1–3, 1 being hardly ever and 3 being often.

### 2.4 Sampling procedure

The purpose of the survey was to develop a baseline, exploratory understanding of material security among LGBTQ+ older adults in the region and was not intended to be representative. Eligibility criteria included being born in or before the year 1970, identifying as part of the LGBTQ+ community, and residing at least part-time in South Texas. South Texas was operationalized using state government definitions (87). We created four subregions based on urban centers where the largest LGBTQ+ organizations in the region exist—Southwest Plains (Laredo-Eagle Pass), Alamo Area (San Antonio), Coastal Bend (Corpus Christi-Victoria), and Lower Rio Grande Valley (McAllen-Brownsville).

After bot detection, removal of incomplete responses, and removal based on age and geographic eligibility criteria, there were 93 survey responses. 10.8% of survey responses were completed in Spanish. The San Antonio town hall had 31 participants and was conducted in-person at an LGBTQ+ senior group. The Rio Grande Valley town hall was virtual, had 8 participants, and was co-sponsored by a local LGBTQ+ organization. Both town halls were 90 min long. Group consensus in both town halls indicated little overlap between survey respondents and participants. 13 participants were recruited for interviews lasting an average of 74 min. Six of the interview participants were recruited from town hall events, while seven reported discovering the study through other recruitment strategies. While town halls included lesbians and transgender women, the proportion of cisgender gay men self-selected to participate in interviews was higher. While Spanish-language interview and town hall protocols were developed and offered, all interviews and town halls were conducted in English.

### 2.5 Quantitative analysis

Analyses were conducted using Stata ver. 19 (StataCorp, College Station, TX) by the second author. Descriptive statistics were calculated for demographic characteristics, which included age, race and ethnicity, sexual orientation, gender identity, identity concordance with birth documentation, citizenship, and the four geographic subregions we designated. Sexual orientation, gender identity, and race and ethnicity were “check all that apply” items. Descriptive statistics were also run for socioeconomic and household characteristics (e.g., health insurance coverage, household size, living alone, homeownership, and housing condition), clinical characteristics (e.g., chronic disease burden, health-related ADL limitation, and self-rated health) and psychosocial characteristics (e.g., being lonely, social support).

Domains of precarity were operationalized using guidance from their sources. Housing, food, energy, technology, and medication precarity were each coded into binary dummy variables (0 = secure; ≥1 = insecure). The variable used to measure housing condition was generated by categorizing apartment/condominium, house, townhouse/duplex, and senior living community options as stable and unstable as rented room, emergency shelter, hotel/motel, permanent supportive housing, trailer/mobile home, and car/RV/boat/other vehicle. Self-reported health was also made into a binary variable in which a response of 0–2 = poor or fair and ≥3 = good/excellent.

Functional limitation was operationalized as 0 if no help needed with activities of daily living (ADLs) or instrumental activities of daily living (IADLs) and 1 if respondents endorsed needing regular assistance with at least one ADL or IADL. An ordinal variable was created for lifetime precarity in which 0 = no lifetime precarity, 1–2 = moderate lifetime precarity, and ≥3 = high lifetime precarity. Chronic disease burden was also converted into an ordinal variable in which 0 = no reported conditions, 1 = one chronic condition, and ≥2 = multimorbidity.

The Social Support Scale was used as a continuous variable with a low score of 0 indicating weak social support and a high score of 20 indicating strong social support. The UCLA Loneliness Scale was also used as a continuous variable, with a score of 0–5 as below the threshold for loneliness and 6–9 categorized as lonely.

The analysis primarily relied on descriptive techniques such as frequencies, percentages, and distributions to characterize the material conditions reported by respondents. Chi-square tests and Fisher’s exact tests were used to test potential correlations between various forms of precarity and LGBTQ+ identity, household characteristics, health characteristics, and social characteristics, respectively.

### 2.6 Qualitative analyses

The research team utilized interpretive description (81) for its established pragmatism in applied research. The goal of interpretive description is to generate a picture of an “ideal future” (Thorne, 2016, p 192), prioritizing applicability over theoretical development. Interpretive description is an inductive analytic method that prioritizes critical interpretation of data and varied thematic verification strategies (19), aligned with data collection processes. Data cleaning and coding occurred iteratively with data collection. Town hall and interview transcripts were de-identified and checked for accuracy by the fifth author. First, third, and fifth authors each memoed while reviewing and open coding transcripts using an ORID (Observational-Reflective-Interpretive-Decisional) protocol, an action learning strategy for focused conversations adapted for research (82) in which each reviewer records their objective observations (what they think the data shows), reflections on their own response, interpretations of meaning, and the decisional impact on collection and analyses. First and third authors then read and discussed memos in concurrence with the review of open codes to assess saturation and guide initial theme development. The second and fourth authors then conducted an audit of initial themes to facilitate theme finalization.

## 3 Results

### 3.1 Participant characteristics

Table 1 outlines survey participant characteristics in full. Demographics of the qualitative sample are further described in Table 2. Demographic information from town hall participants was not formally collected.

**Table 1:** Survey participant characteristics (*n* = 93).

Variables	*n*	%
Demographic characteristics		
Age, mean (SD); range: 55–96 years	61.3 (8.1)	
Race/ethnicity		
Non-Hispanic White	27	29.0
Non-Hispanic Black	22	23.7
Hispanic	34	36.5
Non-Hispanic other^1^	10	10.8
Hispanic heritage (*n* = 34)		
Mexican	26	76.5
Other Hispanic	8	23.5
Gender (non-exclusive)		
Man	43	46.2
Woman	21	22.6
Non-Binary/Gender expansive	21	22.6
Birth ID concordance	65	69.9
Sexual orientation (non-exclusive)		
Lesbian	21	22.6
Gay	26	28.0
Bisexual	38	40.8
SOGI identity group		
Cisgender GBQ men	38	41.3
Cisgender GBQ women	20	21.7
Trans/gender expansive	35	38.0
Citizenship		
US citizen	75	80.7
Other	18	19.3
Region		
Alamo Area	48	51.7
Rio Grande Valley	21	22.6
Coastal Bend	15	16.1
Southwest Plains	9	9.7
Socioeconomic and household characteristics		
Health insurance coverage	71	76.3
Household size, mean (SD); range: 0–8	3.0 (2.0)	
Living alone	13	14.0
House own	43	46.2
Housing condition		
Stable housing	61	65.6
Unstable housing	32	34.4
Health and social characteristics		
Chronic disease burden		
None	17	18.3
One chronic condition	28	30.1
Multimorbidity (≥2 conditions)	48	51.6
Health-related ADL limitation	38	40.9
Self-rated health		
Poor/fair	23	24.7
Good/better	70	75.3
Lonely	63	67.7
Social support, mean (SD); range: 6–20	16.0 (3.1)	

^1^Compiled from respondents who answered American Indian/Alaska Native (AIAN) or Indigenous, Asian/Asian American/, Native Hawaiian/Pacific Islander (AAPI), or Middle Eastern/North African (MENA).

**Table 2:** Interview sample (*n* = 13).

Pseudonym	Age	Race/Ethnicity	LGBTQ+ Identity
Miguel	62	Hispanic	Cisgender gay man
Marie*	74	White	Cisgender lesbian
Marge*	68	White	Transgender lesbian
Arturo*	67	Hispanic	Cisgender bisexual man
Edith	66	White	Cisgender lesbian
Josefina	62	Hispanic	Cisgender bisexual woman
Rafa*	67	Hispanic	Cisgender gay man
Leo*	76	Hispanic	Cisgender gay man
Alan	64	White	Cisgender gay man
Javier	64	Hispanic	Cisgender gay man
Finn*	65	White	Cisgender gay man
Ben	69	White	Cisgender questioning man
James	67	Black	Cisgender gay man

* Participated in a town hall.

#### 3.1.1 Demographics

The average age of the survey respondents was 61.3 years old with a range of 55–96 and a standard deviation of 8.1 years. Over half (51.7%) of the sample lived in the Alamo Area subregion. Latine respondents comprised 36.5% of the sample and among them, 76.5% reported being of Mexican descent. 19.3% of the sample reported a citizenship status other than full citizenship (e.g., permanent resident, visa holder, Deferred Action for Parental Accountability, refugee/asylee status, currently under a withholding of removal, undocumented). 22.6% of the sample. When grouping sexual orientation and gender identity intersectionally, cisgender gay or bisexual men made up 41.3% of the sample. 21.7% of respondents were cisgender women identifying as lesbian, bisexual, or queer. 38.0% of the sample selected a gender identity other than cisgender (e.g., transgender, trans woman, trans man, genderfluid, genderqueer, agender, non-binary, gender non-conforming, two-spirit, or muxe/muxhe).

#### 3.1.2 Socioeconomic and household characteristics

53.8% of survey respondents were renters and 46.2% owned property. 29.0% lived in a home or town home they owned. 22.7% were renting an apartment, condo, or house that was just their household. 22.6% were renting a room in a shared home. 65.6% of respondents reported living in stable housing, while 34.4% reported more unstable conditions.

The mean household size was 3.0 (S.D 2.0, 0.0–8.0). Only 8.6% lived alone (Table 3). Among them, roughly half rented and half owned their housing. The most common household composition among survey respondents (26.8%) was to live with one other person, most commonly a spouse or romantic partner. Those living with three to five other people were likely to live with a romantic partner and either a minor child or child over the age of 18. 16.8% noted they were living with parents or extended family members. 9.7% reported living in a household of six or more and were more likely to be living with non-relatives who were not their romantic partners.

**Table 3:** Household composition, non-cumulative (*n* = 93).

Number of household members	Total	Spouse/Romantic partner(s)	Parents/Extended family	Non-relative(s)	Child under 18	Child over 18
Solo	8.6%	-	-	-	-	-
1 other	26.7%	16	3	3	0	0
2 others	19.2%	15	2	3	0	1
3 others	16.0%	14	2	0	2	6
4 others	11.7%	10	3	0	4	5
5 others	7.4%	5	3	1	3	4
6+ others	9.7%	2	2	9	0	1
		65.9%	15.9%	17.0%	9.6%	18.1%

#### 3.1.3 Health and social characteristics

18.3% of survey respondents reported no diagnosed chronic illnesses. 30.1% reported one, while 51.6% reported two or more. 40.9% reported that their health condition had prompted consistent limitation in ADL/IADLs. Type-II diabetes (34.88%) and hypertension (25.60%) were the most reported medical diagnoses. 10.8% reported having been diagnosed with a substance use disorder, and 17.4% reported being diagnosed with a mental health condition. Despite this, 75.3% self-reported their health as good or excellent. 76.3% reported having some form of health insurance.

The average score on the Social Support Scale was 16.0 (SD 3.1, 6.0–20). This is above 15.0, the threshold used to operationalize strong social support. The average score on the UCLA Loneliness Scale was below the threshold for loneliness at 5.65. However, 67.7% of respondents scored positive on the scale. Crosstabulations (Table 4) indicated that 36.9% reported high levels of actionable support while simultaneously scoring positive for loneliness.

**Table 4:** Crosstabulation of actionable support and loneliness (*n* = 93).

Actionable social support score	Loneliness < 6		Loneliness ≥ 6	
*n*	%	*n*	%
Actionable support ≥ 15	32	33.7%	34	36.9%
Actionable support < 15	3	3.3%	24	26.1%

70% of respondents reported enduring discrimination in the past twelve months. 50% said that they had faced discrimination for multiple identities. Among those who faced discrimination, half attributed it to sexual orientation. 1 in 5 reported age discrimination. 52.4% of respondents of color described racial discrimination. Among transgender, non-binary, or gender expansive respondents, 20% reported gender discrimination.

### 3.2 Occurrence of material precarity

Table 5 describes the prevalence of material precarity within the survey sample.

**Table 5:** Material needs insecurity among survey respondents (*n* = 93).

Variables	*n*	%
Housing		
Housing insecure	54	58.1
Experienced housing discrimination	66	71.0
Use of housing assistance	30	32.3
Food		
Food insecure	83	89.3
Use of food support services	42	45.2
Utilities		
Energy insecure	77	82.8
Medication		
Medication insecure	64	68.8
Cost-related OTC substitution	19	20.4
Cost-related use of home remedies	14	15.1
Use of prescription cost assistance	57	61.3
Technology		
Technology insecure	11	11.8
Use of technology assistance	51	54.8
Lifetime precarity		
None	16	17.2
Moderate/high	77	82.8

#### 3.2.1 Housing precarity

34.4% of respondents endorsed at least one of three forms of housing precarity the survey asked about: (1) worry about paying housing costs (e.g., rent, mortgage, repairs) in the past twelve months; (2) starting to share housing in the past twelve months to reduce housing costs; and (3) spent at least one day without housing in the last calendar year (e.g., eviction, foreclosure, doubled-up rent free, etc.). 3.2% were experiencing literal homelessness at the time of the survey. 88.9% of those living alone reported worrying about housing costs in the last year.

#### 3.2.2 Food scarcity

65.6% of respondents reported running out of food without the financial ability to buy more at least once this past year. 49.5% reported skipping meals to save money and 38.7% reported eating smaller portions to save money.

#### 3.2.3 Energy insecurity

Over three-quarters of the sample said they changed their electricity, gas, and/or water use because they were worried about their utility prices. 64.5% reported being delinquent on utility bills at least once in the past twelve months. 61% said that they were without a utility for at least one day in the past twelve months due to shut-off.

#### 3.2.4 Technology access

Over 87% of respondents reported regular access to the Internet at home, and 98% stated they had daily access to a cellular phone.

#### 3.2.5 Lifetime precarity

82.8% of respondents reported some form of precarity in their lifetimes. The most common response to the lifetime precarity question was food only (17.2%). However, 14.0% reported lifetime precarity across all five domains; 10.8% reported lifetime precarity in four domains.

#### 3.2.6 Formal service utilization

32.3% of the sample reported using a service or program to assist with housing or housing costs. Respondents shared their housing assistance came from emergency rental assistance, housing vouchers, veteran services, and charitable organizations like churches. 45.2% reported using formal support for food provision or to offset the cost of food. Respondents shared that their food support was often Meals on Wheels, Supplemental Nutrition Assistance Program (SNAP) benefits, neighborhood food banks and pantries, and places of worship. 55.9% had used some sort of support service to access technology in the past 12 months. Examples included public libraries, senior centers, and programs that distribute low-cost devices to older adults.

### 3.3 Factors associated with material precarity

Material needs insecurity was highly prevalent across all sexual and gender identity groups, particularly for food and energy insecurity. While some variation was observed across groups, including higher food insecurity among transgender/gender-expansive respondents and higher housing insecurity among cisgender gay/bisexual women, these differences were not statistically significant (Table 6). This insignificance was also observed in chi-square and Fisher’s exact tests run for both sexual orientation (Table 7) and gender identity (Table 8) separately.

**Table 6:** Material precarity (column %) by SOGI groups (*n* = 93).

Material needs insecurity	Cisgender GBQ men (*n* = 38)	Cisgender LGBQ women (*n* = 20)	Transgender^a^ (*n* = 35)	*p*-value^b^
Housing insecure	19 (50.0)	13 (65.0)	22 (62.9)	0.419
Food insecure	33 (86.8)	17 (85.0)	33 (94.3)	0.468
Energy insecure	31 (81.6)	18 (90.0)	28 (80.0)	0.683
Medication insecure	27 (71.1)	14 (70.0)	23 (65.7)	0.958
Technology insecure	4 (10.5)	1 (5.0)	6 (17.1)	0.459
Lifetime precarity	34 (89.5)	17 (85.0)	26 (74.3)	0.259

a Transgender category included any respondents who selected a gender other than cisgender.

b *p*-values were derived from chi-square tests or Fisher’s exact tests, as appropriate.

**Table 7:** Material needs insecurity (column %) by sexual orientation (*n* = 93).

Material needs insecurity	Lesbian (*n* = 16)	Gay (*n* = 22)	Bisexual (*n* = 22)	Multiple^a^ (*n* = 33)	*p*-value^b^
Housing insecure	10 (62.5)	8 (36.4)	14 (68.2)	21 (63.6)	0.126
Food insecure	13 (81.3)	18 (81.8)	21 (95.5)	31 (93.9)	0.280
Energy insecure	14 (87.5)	15 (68.2)	21 (95.5)	27 (81.8)	0.120
Medication insecure	11 (68.8)	11 (50.0)	19 (86.4)	23 (69.7)	0.082
Technology insecure	1 (6.3)	3 (13.6)	1 (4.6)	6 (18.2)	0.464
Lifetime precarity	13 (81.3)	20 (90.9)	20 (90.9)	24 (72.7)	0.257

a The survey item on sexual orientation allowed for multiple responses.

b *p*-values were derived from chi-square tests or Fisher’s exact tests, as appropriate.

**Table 8:** Material needs insecurity (column %) by gender identity (*n* = 93).

Material needs insecurity	Cisgender (*n* = 58)	Not cisgender (*n* = 35)	*p*-value^a^
Housing insecure	32 (55.2)	22 (62.9)	0.467
Food insecure	50 (86.2)	33 (94.3)	0.310
Energy insecure	49 (84.5)	28 (80.0)	0.583
Medication insecure	41 (70.7)	23 (65.7)	0.649
Technology insecure	5 (8.6)	6 (17.1)	0.320
Lifetime precarity	51 (87.9)	26 (74.3)	0.154

a *p*-values were derived from chi-square tests or Fisher’s exact tests, as appropriate.

Similarly with regard to self-reported health, more lesbian respondents reported being in fair health and less likely to respond “good” or “very good” when doing comparative analyses, but these differences were not statistically significant and being a lesbian was not significantly associated with specific health conditions or needing assistance with daily living (ADLs).

In adjusted models (Table 9), a larger household size was associated with higher odds of housing, energy, medication, and lifetime material precarity. Loneliness was strongly associated with housing and energy precarity, while greater social support was protective against food precarity. Having one or more chronic conditions was significantly associated with lifetime material precarity, although confidence intervals were wide. Other factors, including housing instability and self-rated health, were not significantly associated with material precarity domains.

**Table 9:** Factors associated with different forms of material precarity (*n* = 93).

	Material precarity, AOR (95% CI)					
Variables	Housing	Food	Energy	Medication	Technology	Lifetime
Household size	1.46 (1.06, 1.97)*	2.15 (0.99, 4.65)	2.40 (1.28, 4.51)**	2.07 (1.35, 3.16)**	0.64 (0.33, 1.24)	1.84 (1.10, 3.10)*
Housing condition (stable housing)						
Unstable/precarious housing	2.74 (0.93, 8.11)	4.20 (0.42, 42.44)	0.68 (0.13, 3.58)	2.39 (0.68, 8.47)	0.84 (0.13, 5.37)	3.69 (0.55, 24.95)
Chronic disease burden (none)						
One or more chronic condition	1.58 (0.39, 6.35)	1.00 (0.08, 12.50)	2.95 (0.40, 21.89)	0.96 (0.18, 5.19)	0.61 (0.06, 6.38)	16.56 (2.32, 118.06)**
Self-rated health (good/better)						
Poor/fair	2.05 (0.60, 7.02)	0.66 (0.10, 4.46)	1.53 (0.22, 10.67)	3.76 (0.84, 16.77)	0.44 (0.04, 4.47)	3.66 (0.35, 38.10)
Lonely (no)						
Yes	3.85 (1.29, 11.52)*	1.00 (0.17, 5.83)	11.84 (2.18, 64.31)**	1.70 (0.52, 5.53)	3.25 (0.32, 33.60)	1.86 (0.36, 9.71)
Social support	0.88 (0.73, 1.06)	0.59 (0.36, 0.95)*	0.87 (0.64, 1.19)	0.91 (0.73, 1.12)	1.03 (0.74, 1.42)	0.91 (0.64, 1.30)

AOR, Adjusted Odds Ratio; CI, Confidence Interval; the reference group is in parentheses. Statistical significance indicated by *p < 0.05; **p 0.01; ***p < 0.001.

### 3.4 Qualitative themes from town halls and interviews

Qualitative findings from town halls and interviews provided greater depth of insight and collective hypothesizing surrounding quantitative findings. Thematic findings from qualitative data collection included:

#### 3.4.1 LGBTQ+ affirming affordable senior housing

Housing emerged as the paramount concern within town halls and interviews. Twelve of the thirteen interviewees explicitly identified LGBTQ+-affirming affordable senior housing as a desirable long-term goal of localized research. Drivers for this goal were multidimensional. One reason some participants felt LGBTQ+ inclusive housing for older adults was needed was a lack of belonging in existing local options. Marie shared how her peer networks have been impacted by discrimination and the risks of discrimination in senior living:

I know a number of older women who have gone back in the closet when they are in assisted living. They have cut ties with me because they are afraid somehow that I’ll magically out them, which I would never do…I had lunch with a woman, and I wasn’t talking loud, and I said something-- I used the word “gay” in conversation. And her eyes got big and she shushed me, like “what in the world is wrong with you?” You know, “we cannot talk about that here.” I was shocked. And it made me understand how fearful she is.

Marie’s vicarious experience watching other women refrain from disclosing their sexuality made aging-in-place her only perceived option. Another interviewee, Arturo, shared that he had also contemplated a move into a continuum of care but felt a cultural mismatch:

I looked into a couple of the senior homes, I actually went to a couple who said you should come and meet us. I did not feel comfortable, I did not feel acknowledged. I’m not going to change the way I dress, I’m not going to change the way I talk. It did not feel right.

While some had been disappointed by their active consideration of relocation options, others discussed more aspirational visions for their future. These visions were often aligned with social housing. For example, Alan discussed how he and his friend group dreamt about their futures as solo agers:

The other day I heard a radio piece about older people becoming the most active group searching for roommates, and it’s because they are getting to be at an age where they thought they could afford it, but they cannot afford their mortgage or their bills. So they have to take in a roommate. And just how, what a change it is. It’s one of the things we joke about my group, because we are all singles. We’ve all been single for so long, we are so set in our ways. Do we really think we can deal with that? So that’s why we have to win the lottery, so we can build 4 little houses. Or we’ll build a big center house, we’ll have wings, we each have our own wings. We laugh about it, we drive by houses like “Oh my God, look, that looks like a big house, it looks like it has wings for each of us.”

Alan imagined an affordable housing environment where he and his friends had privacy but could be in proximity with one another. Similarly, Miguel was plotting an exit from a skilled nursing facility during the time of his interview. He continued to live there after being evicted during his stroke hospitalization, despite not needing the level of care. He spoke about his care needs and budget, hoping for a homeshare:

Me and a buddy here want to move out, and we are trying to figure out how to go about that. A 2-bedroom apartment. It’s gotta be gay friendly. Do not have to be a gay man, but gay friendly. Heck, when I had my own apartment, I had a roommate. He wasn’t gay. I had little gay trinkets out and about, you know. Yeah, my house is like “I’m gay.”

In Miguel’s navigation of what his future housing could look like, he emphasized that while shared identity wasn’t a priority, home meant expressing his identity as a gay man. Like Miguel, other interviewees shared that a history of communal living made the idea of homesharing acceptable, if it was LGBTQ+ affirming.

Another factor that arose in conversations about LGBTQ+ affirming social housing for older adults were the unique regional attributes driving LGBTQ+ geographies and migrations. Many participants spoke of deep pride in the region and Tejano cultural ties to place. Nine of the thirteen participants had been born and raised in the region, and some had stayed throughout their entire lives. Others left but chose to return to the region:

Leo: I went, why am I paying all this rent here? Let me just go back. My mom was living here and my brother, they were all here. I remember my sister told me, oh come back home, there’s no lines, there’s no traffic. I mean in L. A., I lived 16 miles from my job, and it would take me two and a half hours to get to work.

Javier: My parents were getting older and I said, you know what, I’m just gonna be there, closer to them. For the last 10, 15 years of their lives. It’s been a couple decades, though-- I find San Antonio to be somewhat of a hidden gem. It’s a wonderful place. I love the culture here, the art scene. It’s got some deep entrenched economic poverty issues that have long gone unaddressed, that probably coincide with some racism and class issues. It’s got some challenges, some problems, but it’s got a lot of benefits.

While both Leo and Javier’s families were part of their decisions to return, Leo explained how when it came time for him to retire, he chose to return to San Antonio for an easier way of life. Javier expressed attachment to the local community and went on to describe how through active civic engagement, participates in addressing poverty and racism in the neighborhood he grew up in.

Others spoke of how South Texas was a retirement destination and how this shapes the need for LGBTQ+ affirming senior housing options. Marge, a transgender woman previously living in West Texas, shared one of the primary reasons for her relocation to the region:

I was going to support group meetings like, in Dallas and Albuquerque…a five hour to eight hour drive either way to go to a support group meeting. One way. And so we made the decision, said “okay, let us get the house ready to sell and move.”

Marge and her partner relocated to the region to have increased access to LGBTQ+ affirming services and community. In the Rio Grande Valley town hall, participants noted that weather also drives part-time residents to the region, including LGBTQ+ older adults. This was also identified as a source of community tension and differences in programming needs:

Usually white, retired, middle class to upper middle class spend half the year here. And they have a significant impact on parts of the Valley and the economic landscape… people may retire here from places like San Francisco or even places like Dallas and Houston. They have very specific ideas of what queer organizing and queer community and queer visibility need to look like. It clashes with some of the older folks who have chosen to or ended up making their lives here.

Town hall participants went on to discuss seasonal differences in “Winter Texan”-populated cities and towns, indicating that parts of the region may be more impacted by seasonal fluctuations in population and the sociopolitical dynamics that migration may drive. LGBTQ+ senior housing needs may need to be responsive to cultural and socioeconomic differences.

Other town hall participants and interviewees noted that recent legislative violence against queer and transgender people have prompted them to consider crossing the border. In the words of Edith:

I got my temporary residence in Mexico this last year just because of what’s going on in the country and my concern for what’s going on in the country and some concern about you know if I needed to exit how would I do so. One of my exit plans would be Mexico.

While Edith spoke of transnational migration as a solution to a yet-to-be-crossed threshold, Josefina shared that this trend was already visible in her life:

Three or four people that I know have moved out of San Antonio and moved to other locations because they are worried about security as far as their lives. They do not want to have to be worried about being attacked or anything like that. They’re worried about finances. They owned homes and they sold them or are renting them out because the economy in the United States is just going downhill and the health part is also going downhill.

Josefina’s observations indicated how complex and intersecting political and structural forces drove LGBTQ+ older people in her networks out of the region. Participants who spoke of political violence as a push factor for migration away also discussed the protective benefits of having safe housing and gathering places in the region.

#### 3.4.2 Health justice efforts to address LGBTQ+ disparities

Another convergence between town hall, interview, and survey data was in health disparities. While superficially, healthcare coverage and costs may appear to be concerns of older adults in the United States regardless of sexual orientation and gender identity, some interviewees were able to articulate differential impacts of these costs in the trajectories of their health and economic well-being, in part due to social capital. Rafa shared that two major life events– the termination of a long-term relationship and multiple recurrences of prostate cancer shortly thereafter– caused a major economic and social fallout that was hard to recover from. He shared that he assumed that as a gay man, he did not have legal rights in the separation and left the relationship with “nothing.” Now living in a 250-square foot accessory dwelling unit prone to scorpions in the rural outskirts of San Antonio, Rafa stated:

It took me nine months to get a biopsy because I did not have anybody to go with me for the biopsy because I needed someone… it’s not just us, because there’s a lot of groups that need that help, but I’m saying what concerns me, but if you get an illness like I did, on this last cancer, I had to pay $13,000 because my insurance did not cover it. Why do I go to the doctor? I will have to pay this and that and the other and they always want a credit card. I do not have a credit card.

Structural violence prohibited Rafa from exercising his legal rights, positioning him with a deficit of material and social resources. Rafa shared that his access to care was delayed due to his geographic and social isolation, which then impacted the progression of his disease when he finally did access care. He also shared how previous interactions with healthcare institutions that centered profit over patient care also informed his reluctance to receive medical care. Cumulative disadvantages faced by LGBTQ+ older adults also arose in town halls. In the San Antonio town hall, a white gay man in his late 60s expressed a tension:

It’s important to make it clear the difference between those of us who came out young and faced a lifetime of being gay or living through AIDS. Some people came out in their 40s or 50s or even more recent and their aging might be easier, you know? If they had two incomes or have kids to support them or did not get discriminated against in the workplace.

This declaration resonated with town hall participants, who continued to discuss how turning points like “coming out” changed economic and health trajectories. Later, a cohort of Latine participants discussed how racism, xenophobia, and colorism informed LGBTQ+ life course trajectories.

Healthcare discrimination and fear of self-disclosure was extensively discussed in the San Antonio town hall by both cisgender and transgender participants. Transgender town hall participants noted specific risks around mammography and reproductive health. One transgender woman in the town hall noted that identifying as transgender on clinic forms can be a “magnet for whoever is looking” and that she’d recorded her genital reconstruction surgery as a hysterectomy on paperwork. Other town hall participants anchored these safety precautions in the current political landscape in the state. One gay man, who shared his HIV-positive status with the group, expressed:

I’m not going to lie, but I’m also not going to come out in my doctor’s office because I do not know where they are at with some of that stuff, with all this retraction of DEI programs.

In interviews, two participants brought up the value of having affirming and safe healthcare professionals in their lives. Marge, the transgender woman interviewed, noted that leaving a gender-affirming doctor in her last place of residence was one of the hardest aspects of relocation.

#### 3.4.3 Social programming for LGBTQ+ older South Texans

Across the data, older adult participants clearly articulated programming needs. Following housing, the local improvement most recommended in town halls and interviews was social programming and community space. Convergent with survey data indicating relatively strong instrumental support, interviewees discussed how informal mutual aid and neighborhood exchange offset housing costs. Leo shared that in his neighborhood, he can get lawn maintenance and basic home repair assistance from neighbors for a low price. Town hall participants in San Antonio showcased a mutual aid table where people donate household items and food, or barter and trade with one another. One participant reflected how it reduced her weekly grocery bill. Another participant, a woman experiencing homelessness, shared she took some things from the group to furnish her last apartment.

For some interviewees, the LGBTQ+ senior group in San Antonio extended beyond instrumental support. Rafa, who had been a member of the LGBTQ+ senior group for about three years prior to his interview, reflected on life before attending:

I was very depressed, going down the rabbit hole. I was going down; I had suicidal thoughts. Once I got there [LGBTQ service], it was just like even with all the stupidities and nonsense, I loved it. They were my peers, they were gay. 15–20 people!

Rafa discovered the group through word of mouth from another older gay man at a local cruising spot, an encounter that Rafa described to the interviewer as a “literal life saver.” Interviewees who were not recruited through the LGBTQ+ senior group often expressed a desire for a low-structure, peer-led socialization and mutual aid group for LGBTQ+ older adults.

Age discrimination and age segregation was a recurring barrier to addressing social isolation among interview participants. Even for those who were heavily involved in social groups, volunteerism, and informal networks of family and friends, loneliness was still part of their day-to-day experience. Ben, a retired volunteer at a local LGBTQ+ service organization, shared:

I still experience a certain amount of loneliness myself… there is a side of me that my spouse has never really seen. There’s a certain need for fellowship, and for me, attending [volunteer organization] fills that void. For people who do not have this option, it would be so tough. I volunteered with another group and there were some people my age, but not many. I would go to events with them and everything, and they would just ignore you. I’ve tried to strike up conversations but it’s like, what do you talk about with a 20-year-old person?

For Ben, age segregation and age stigma were barriers he needed to bypass to find a civic engagement space that adequately addressed his loneliness. Nearly all interviewees discussed a link between loneliness and a disenfranchisement around their contribution as an elderly to the queer community. Another interviewee, James, who also volunteered in service of LGBTQ+ and HIV-affected communities similarly declared that LGBTQ+ community groups needed to improve how they “facilitate treating older people like human beings.” Both Ben and James discussed living through the HIV/AIDS epidemic and current-day legislative rollbacks as motivators for giving back to the community, particularly LGBTQ+ youth and emerging adults. Arturo expressed that because he never had children of his own, there aren’t enough opportunities for him to offer mentorship and guidance to the next generation. He told a story about supporting his nephew through his first heartbreak, acknowledging that it may be meaningful and necessary for LGBTQ+ youth to have LGBTQ+ elder mentors in navigating healthy relationships. Other examples of wise advice for younger generations that emerged from interviews were from Marge, a highly educated transgender woman with multiple practical skill sets, who encouraged young people to develop in-demand skill sets to offset the risk of job discrimination. Javier gave a personal anecdote to illustrate how LGBTQ+ older adults could support LGBTQ+ emerging professionals in finding work-life balance and break away from the “overachiever” archetype that LGBTQ+ people can internalize. Multiple interviewees shared that if they could give advice to young people, they would encourage them to learn in-person communication skills and find strategies for meeting new people offline, including Finn, who also declared a “worry about archiving and preserving history”,

Marie and Finn, who both had leadership experience in community advocacy and organizing during the HIV/AIDS epidemic, both raised concerns about their generations’ legacy and the possible consequences of social movements not anchored in history. Finn told anecdotes of how he and his now-deceased partner participated in health and social care for people dying of AIDS-related illness in the late 1980s and early 1990s, sharing how non-profits emerged and devolved over the years. Marie shared history of the LISA (Lesbians in San Antonio) hotline, saying:

It’s shocking to me that everybody keeps trying to recreate the wheel. I do not think it’s unusual, that kind of tension between older and younger activists, but know your history.

In the Rio Grande Valley town hall, attendees mirrored this frustration about age segregation’s impact on surviving the current political landscape. One participant shared:

It’s important to take into account what the older members of the LGBT community— what we have gone through, you know? We’re not old and senile, we still have a lot of information that we can share. I think just being included and remembered for that is something I hope the younger folks consider or remember.

Six of the thirteen interviewees spoke about adopting caregiving roles in middle age. In these conversations, interviewees spoke about the uniqueness of being an LGBTQ+ caregiver, like Marie:

My older sister lived with me for two and a half years. I brought her down because she was not being treated well where she was living, and her health was very fragile… frankly, that caregiving has been a drain on some of my money. And I also think it prevented me from maybe having some of the social relationships I would’ve liked to have, but it was right there in front of me, I felt it was my place to do it. And lesbians, we are so damn competent. I back away from that now because frankly, I need the energy for myself.

Marie noted that part of her caregiving experience was reluctant adoption, and that choosing to do so involved sacrifice. Marie identified benevolent sexism and homophobia in cultural images of lesbians as endlessly capacious for labor. She later discussed how being childless and without a legible long-term partnership opened the door for adopting a caregiver role. Similarly, Edith discussed sacrificing leaving San Antonio for a “more gay-friendly city” to take on caregiving for her aging parents, and that this continued to be a “what-if” in her current life. For some interviewees, like James, caregiving was a gift:

My mom just died a year and a half ago, so we had her for like, 12 or 13 years. And we had so much fun. My mom was one of those people who put everything into her kids and grandkids…after my dad passed, I started dressing her, and we stepped out. Let me tell you, she was it. My husband and I, we just really had a time with her, it was really special.

In James’ story of caregiving for his mother, he discussed the healing benefits of inviting his mother into the home he shared with his husband, and how caring for her was an expression of queer joy. Regardless of interviewee relationships with caregiving, the particularities of LGBTQ+ caregiving were reflected.

## 4 Discussion

While the findings of this mixed methods study largely align with existing empirical data on LGBTQ+ aging in the rural U.S. South that identify health disparities, the lack of healthcare protections, discrimination and stigma, and gaps in community support (20), findings also highlight distinct vulnerabilities faced by LGBTQ+ older adults across the South Texas region. Quantitative survey data indicated that material precarity was widespread among older LGBTQ+ South Texans, especially food, energy, and medication. Precarity was socially patterned, with household size, loneliness, social support, and chronic disease burden emerging as important factors.

Current and historical housing precarity’s influence on material and social well-being was a major finding. Household size was associated with current precarity in several domains as well as lifetime precarity. Loneliness was strongly associated with housing and energy precarity. Together, these findings speak to housing’s role in the health and well-being of LGBTQ+ people throughout the life course. A larger household size may reflect shared economic vulnerability rather than resource pooling, particularly in the context of chosen family networks. While among racially minoritized populations, cohabitation among extended family and social networks may be both culturally normative and protective against material precarity and social isolation (21, 22), familismo can simultaneously reinforce cishetero normativity (23) and be overaccentuated as a resilience factor among Latinx LGBTQ+ adults (24). Narrative tropes of familismo may also serve to justify frontier logics and settler colonialism that drive overcrowding, poor housing quality, and displacement (25). This may also connect to the nuanced caregiving experiences that many interviewees discussed adopting, as caregiving among LGBTQ adults has indicated greater financial (26) and social–emotional strain (27) than heterosexual and cisgender counterparts. Loneliness was strongly associated with housing and energy precarity, underscoring the role of social isolation and sense of belonging in compounding economic hardship.

Our findings align with gerontological theorization of cumulative advantage and disadvantage (CAD) throughout the life course, in that intersecting cumulative disadvantages– driven by structural, disciplinary, cultural, interpersonal, and intrapersonal power– produce health and economic inequities across the life course for minoritized older adults (28). Chronic disease burden was linked to lifetime material precarity, consistent with CAD across the life course. Current unstable housing conditions were also linked to lifetime material precarity. Town hall and interview participants articulated how key historical events like the HIV/AIDS epidemic, recent federal elections, and enactment of state legislation shifted life both longitudinally and in the present, aligning with theorization that life course impacts of unjust policy and the accumulation of social and economic disadvantage are then amplified by ageism and age-targeted policies that amplify inequalities in later life (29). Participants described how life trajectories, even within the community, differed based on personal and interpersonal turning points that either generated vulnerability to or insulation from structural violence. This is aligned with recent research applying life course theory to older gay men (83), which has emphasized the need for both expansion of theory beyond “chrononormativity” (30) but also intracohort variation among older gay men. While there are clear parallels between literature explicating the Latino Health Paradox and queer theory on the protective role of intracommunity support against minority stress, little knowledge has been empirically established about intersectional sociocultural resilience among LGBTQ+ Latine people across the life course (13, 31). Further research must be done to understand “chosen *familismo*,” spatial displacement related to immigration and queerness, and its impact on health and mortality for LGBTQ+ Latine older adults.

In the sample, formal service utilization was high, as was actionable social support. This aligns with existent research that suggests LGBTQ+ adults use safety net programs such as SNAP, SSI/SSDI, and housing assistance more than cisgender heterosexual counterparts (4). Qualitative findings indicated that intracommunity support occurred between LGBTQ+ older adults, as well as between LGBTQ+ older adults and their broader social networks. This finding bolsters exploratory research in other regions of the U.S. South (32) that have also identified intracommunity support as a protective factor for LGBTQ+ older adults. Actionable social support appeared protective against food insecurity, suggesting that social networks in LGBTQ+ older adults’ lives buffer hunger and food hardship more effectively than other kinds of hardship. This also may point to the success of congregate meal programs, face-to-face meal delivery, or other senior nutrition interventions with social components.

However, loneliness was present in roughly two-thirds of the sample, and still pervasive among a subgroup of respondents who reported having average or above average actionable social support. Thematic findings from town hall and interview data pointed to age segregation, care burden, and lack of spaces as barriers to meaningful social connection. Social programming solutions such as peer-led socialization groups for LGBTQ+ older adults, LGBTQ+ caregiving, and intergenerational programming to support legacy, mentorship, and the advancement of LGBTQ+ rights movements were recommended by participants. Qualitative findings emphasized the value of community gathering spaces with emplaced activities and services, such as LGBTQ+ affordable housing sites and LGBTQ+ health sites. Given the established care gaps within formal mental health services for LGBTQ+ people in the region (33), it is a logical assumption that increased specificity of services, peer-led services, and services in a designated queer space are desired. Because emerging adulthood and later life are when social connectedness is most at risk (34), intergenerational programming may also be synergistic with local and regional efforts to support LGBTQ+ youth.

Survey respondents who reported loneliness were statistically more likely to report housing and energy precarity. The number of people in one’s household did not significantly explain this connection, suggesting there may be a more direct link between current precarity and the experience of feeling socially isolated and excluded. In meta-analysis, loneliness has shown to be higher in LGBTQ+ adult samples than cisgender and heterosexual counterparts (35). Loneliness and social isolation are established risks among LGBTQ+ older people (36), in part due to experiences of lack of belonging and forced invisibility (37, 38). Lifetime experiences of discrimination may result in social inhibition, internalized homophobia and transphobia, and/or the development of less prosocial skills (39, 40), though some research has suggested a waning impact over the life course (41). Experiencing political violence has been correlated with loneliness outcomes, given that it communicates unwantedness and structural lack of care, but this has also been shown to be mediated by intracommunity belongingness (42).

### 4.1 Implications

Collectively, these findings suggest that material precarity among older LGBTQ+ South Texans reflect the interplay of structural economic strain, health burden, and social connectedness. Material precarity and negative social outcomes like loneliness are governed by interconnected, not siloed, systems. Interviews and town halls illustrated this interplay, showcasing how policies, organizational bureaucracies, cultural norms, and interpersonal dynamics can perpetuate cumulative disadvantage through the life course. At the same time, this study’s findings revealed personal and community-level strategies for persevering in the face of sociopolitical challenges, including clear pathways for future research, advocacy, and programming.

Formal and informal support systems matter, but existing services may not be sufficient, accessible, or affirming for older LGBTQ+ South Texans. The expansion of LGBTQ+ affirming housing, social programming, and healthcare in the region may both address formal care gaps, as well as foster a strengthening of informal support. Creating a localized hub for LGBTQ+ service organizations where healthcare, social services, community organizations, and housing could be co-located would increase resource awareness, spur intergenerational contact, support accessibility and retention among service users, and create a more financially sustainable service model in an increasingly scarce funding environment. Particularly in times of increased state-sanctioned violence, small localized “fractal” practices can drive regional transition (43).

Multiple models for affordable LGBTQ+ senior housing exist across the country (44). In Texas alone, Dallas, Austin, and Houston all boast LGBTQ+ affordable senior housing (45–47). Affirming and safe social housing for LGBTQ+ older adults offers social and environmental solutions that have been shown to reduce loneliness and perceived disconnection from the larger LGBTQ+ community (48), as well as have iatrogenic health benefits (49). LGBTQ+ centered homeshare programs may offer a culturally congruent, scalable solution to LGBTQ+ older adult housing precarity; not only is homesharing a cost-saving measure but aligns with queer survival and solidarity histories.

Past studies indicate that everyday symbols of inclusivity like flags, stickers, language, and clothing, can provide simultaneous affirmation and dis-ease, shaping trans and gender diverse people’s identity, emotions, and access to support (84). Symbolic representation without affirming policies or competent professionals is not satisfactory. Expanding local partnerships with national networks, such as SAGECollab (50), could provide non-profits and municipal government agencies in the region with necessary education, training, and consultation. Area Agencies on Aging and other community-based organizations that primarily focus on serving older adults can also contract with and coordinate alongside local LGBTQ+ organizations to offer skills and knowledge transfer and mobilize community action.

While scholars have begun to test emergent interventions and program models for addressing social isolation (51) and promoting cardiovascular health (52, 85) among older LGBTQ+ populations, future research should prioritize continuing to adapt and evaluate the impact of existing best practices. There is a role for community-engaged research to inform, facilitate, and evaluate cross-industry partnerships working to address service gaps for LGBTQ+ older adults. Given that many participants expressed exclusion from LGBTQ+ community spaces due to age bias, and that participants also emphasized the importance of eldership and legacy in the current sociopolitical climate, researchers can support the integration of local artists, activists, and organizers in public health interventions that focus on oral history, archival preservation, and intergenerational exchange (53). Radical intergenerational alliances have been the bedrock of anti-ageism movements like the Gray Panthers (54), are impactful among LGBTQ+ chosen families (55), and can continue to be a useful strategy for healthcare access, housing rights, and other ageless concerns.

### 4.2 Limitations

The survey was open for a long period of time due to difficulties with recruitment, funding, and geographic spread. Sample size and spread inhibited possible inferential analyses. While there were planned town halls in Corpus Christi and Eagle Pass, the research team could not feasibly conduct them after loss of funding. This resulted in recruitment being far more robust and successful within Bexar County and surrounding areas than in the rest of the region. Cisgender women, transgender people, and Black Texans were not robustly represented within interview data. Future team-based research in South Texas with LGBTQ+ older adults can benefit from a team inclusive of underrepresented subgroups, including deeper engagement with older South Texans from these groups as co-researchers. With more resources, future research may be able to implement a respondent-driven sampling strategy as best practice to ensure both a larger sample and greater sample diversity (86). Qualitative content on healthcare revealed how the operationalization of health precarity in the survey design was not inclusive of cost barriers to healthcare beyond prescriptions. Future surveys could benefit from drawing upon standardized questions on cost barriers in healthcare utilization more broadly, such as those in the Behavioral Risk Factor Surveillance Survey (56).

## 5 Conclusion

This study’s findings underscore how ageing experiences for older LGBTQ+ South Texans are shaped by historical and ongoing structural violence that drives precarity in basic human rights such as housing, food, healthcare, and social connection. However, analyses also indicated profound individual, interpersonal, and community-level resilience. Through a community-engaged approach, older LGBTQ+ South Texans voiced a clear vision for culturally responsive, LGBTQ+ affirming interventions not only to secure those who are currently aging, but future generations. Affordable housing models designed with LGBTQ+ older adults in mind do more than provide shelter—they can serve as a fractal hub that reduces the exposure to and impact of interpersonal discrimination and systemic oppression. When an intergenerational community space can be built around affordable, inclusive senior housing, the entire community benefits from its health and social benefits. Future gerontological research and public policy must prioritize strategies that honor the unique life course trajectories of LGBTQ+ older adults in the region, many of whom with deep connection to South Texas as a place and Tejano culture and identity. Policymakers and practitioners are urged to invest in community-engaged solutions that center housing solutions with emplaced health and social services that can not only protect LGBTQ+ older adults but honor their vital role as elders in our communities at large.

## Data Availability

The raw data supporting the conclusions of this article will be made available by the authors, without undue reservation.

## References

[1] Grenier A Hatzifilalithis S Laliberte-Rudman D Kobayashi K Marier P Phillipson C. Precarity and aging: a scoping review. Gerontologist. (2020) 60:620–32. doi: 10.1093/geront/gnz135, 31675418

[2] Hernandez M Markides KK Cantu P. The effect of financial strain on the health outcomes of older Mexican-origin adults: findings from the Hispanic established population for the epidemiological study of the elderly (H-EPESE). Int J Aging Hum Dev. (2024) 99:3–24. doi: 10.1177/00914150241231187, 38354308 PMC11295423

[3] Bhat AC Fenelon A Almeida D. Housing insecurity pathways to physiological and epigenetic manifestations of health among aging adults: a conceptual model. Front Public Health. (2025) 13:1485371. doi: 10.3389/fpubh.2025.1485371, 39916715 PMC11799248

[4] Henderson ER Hotchkiss M Lin CAC Blosnich JR. Material hardship and use of social safety net programs among LGBT adults and their families. Anal Soc Issues Public Policy. (2025) 25:e12432. doi: 10.1111/asap.12432, 40734955 PMC12303088

[5] Glick JL Lopez A Pollock M Theall KP. Housing insecurity and intersecting social determinants of health among transgender people in the USA: a targeted ethnography. Int J Transgender Health. (2020) 21:337–49. doi: 10.1080/26895269.2020.1780661, 34993513 PMC8726680

[6] Adan M Schribani M Gadomski A. Worry and wisdom: a qualitative study of transgender elders’ perspectives on aging. Transgender Health. (2021) 6:332–42. doi: 10.1089/trgh.2020.0098, 34993305 PMC8664103

[7] Williams Institute. (2023). “Data in Review.” Available online at: https://williamsinstitute.law.ucla.edu/wp-content/uploads/Williams-Institute-Data-in-Review-2023.pdf

[8] Mallory C. Brown T. N. T. Russell S. Sears B. (2017) The impact of stigma and Discrimination against LGBT People in Texas Williams Institute. Available online at: https://williamsinstitute.law.ucla.edu/wp-content/uploads/Impact-LGBT-Discrimination-TX-Apr-2017.pdf (Accessed June 25, 2026).

[9] Transgender Education Network of Texas. “89th Regular Session: The Overview.” (2025). Available online at: https://transtexas.org/bill-tracker/ (Accessed August 22, 2026).

[10] Texas Pride Impact Funds. (2018). IMPACT! Texas Statewide LGBTQ Community Needs Assessment Texas Pride Impact Fund. Available online at: https://texaspridecf.org/impact-texas-needs-assessment/ (Accessed June 25, 2026).

[11] Oswald A Cooper L. Addressing equity and justice in age-friendly communities: considerations for LGBTQ+ older adults of color. Gerontologist. (2024) 64. doi: 10.1093/geront/gnae050, 38767047

[12] Riosmena F Everett B Rogers R Dennis J. “Negative acculturation and nothing more?” cumulative disadvantage and mortality during the immigrant adaptation process among Latinos in the United States. Int. Migration Rev. (2015) 49:443–78. doi: 10.1111/imre.12102, 28104925 PMC5241135

[13] Ruiz JM O’Neill RM Juster R-P Irgens MS. "Dimensions of diversity and influences on health disparities". In: Schneiderman N Smith TW Anderson NB, editors. APA Handbook of Health Psychology, Volume 1: Foundations and Context of Health Psychology, vol. 1. Washington, D.C: American Psychological Association (2025)

[14] Greenwood D. Pragmatic action research. Int J Action Res. (2007) 3:131–48. Available at: https://nbn-resolving.org/urn:nbn:de:0168-ssoar-412899

[15] Berkowitz SA Meigs JB DeWalt D Seligman HK Barnard LS Bright O-J . Material need insecurities, control of diabetes mellitus, and use of health care resources. JAMA Intern Med. (2015) 175:257–65. doi: 10.1001/jamainternmed.2014.6888, 25545780 PMC4484589

[16] Freedman VA Schrack JA Skehan ME. “National Health and Aging Trends Study User Guide: Rounds 1–14.” Final Release. Baltimore: Johns Hopkins Bloomberg School of Public Health (2026).

[17] Chung GC Marottoli RA Cooney LM Jr Rhee TG. Cost-related medication nonadherence among older adults: findings from a nationally representative sample. J Am Geriatr Soc. (2019) 67:2463–73. doi: 10.1111/jgs.16141, 31437309

[18] Hughes ME Waite LJ Hawkley L Cacioppo JT. A short scale for measuring loneliness in large surveys: results from two population-based studies. Res Aging. (2004) 26:655–72. doi: 10.1177/0164027504268574, 18504506 PMC2394670

[19] Wilson J. Interpretive description and reflexive thematic analysis: exploring conceptual coherence and methodological integrity. Qual Health Res. (2025) 1-13. doi: 10.1177/10497323251378303, 41060688

[20] McKay T Lampe N Barbee H Prasad A Gonzales G. An evidence-based framework for supporting older LGBTQ+ adults in rural communities: findings from the LGBTQ+ social networks, aging, and policy study. Public Policy & Aging Report. (2024) 34:150–3. doi: 10.1093/ppar/prae019, 39734580 PMC11670641

[21] Agrawal V Al Snih S. Living arrangements and cognitive impairment among Mexican American older adults: nativity differences. J Appl Gerontol. (2025):07334648261422904. doi: 10.1177/07334648261422904, 41637721 PMC13010988

[22] Mahaffey D. Reclaiming collective strength: the untapped power of multigenerational living in black American families. J Forensic Soc Work. (2025) 9:21–41. doi: 10.15763/issn.1936-9298.2025.9.2.21-41

[23] McCandless-Chapman O Ottaway A Stone AL Robinson BA. Latinx LGBTQ+ youth and grandparents: intergenerational solidarity, precarious familismo, and cisnormativity. J Marriage Fam. (2024) 86:614–32. doi: 10.1111/jomf.12979

[24] Alvarez-Hernandez LR Villalobos BT Hernandez Rodriguez J Logan C Mena Teran J Estrada Gonzalez SM. ‘It was basically don’t say gay with a therapist face’: LGBTQ+ Latinx individuals’ experiences with mental health services in South Texas. Psychol Sex Orientat Gend Divers. (2026). doi: 10.1037/sgd0000904

[25] Launius S Boyce GA. More than metaphor: settler colonialism, frontier logic, and the continuities of racialized dispossession in a southwest U.S. City. Ann Am Assoc Geogr. (2021) 111:157–74. doi: 10.1080/24694452.2020.1750940

[26] Anderson JG Flatt JD. Characteristics of LGBT caregivers of older adults: results from the National Caregiving in the U.S. 2015 survey. J Gay Lesbian Soc Serv. (2018) 30:103–16. doi: 10.1080/10538720.2018.1440681

[27] Hall S Hubbard Murdoch N Dyck D Stacey A. Queer caregiving with older adults: a scoping review of the lived experiences of LGBTQ+ family and friend caregivers. Soc Sci Human Open. (2025) 11:101324. doi: 10.1016/j.ssaho.2025.101324

[28] Perone AK Zhou L Coldon T Solorio M Paget A Osborne A. Equitable aging in health framework: a multi-systems and multi-level approach to health challenges and supports for transgender older adults. Innov Aging. (2025) 9. doi: 10.1093/geroni/igaf103, 41220809 PMC12598661

[29] Dannefer D Han C. "Process, policy, and unintended consequences: the life-course patterning of cumulative (dis)advantage". In: Wilmoth JM London AS, editors. Life Course Implications of US Public Policies. New York, NY: Routledge (2021). p. 17–31.

[30] Baars J. Critical turns of aging, narrative and time. Int J Ageing Later Life. (2012) 7:143–65. doi: 10.3384/ijal.1652-8670.1272a7

[31] Flores Y. Chicane Mental Health: Alma, Mente, y Corazon. 2nd ed. Tucson, AZ: University of Arizona Press (2026).

[32] Dakin EK Williams KA MacNamara M. Social support and social networks among LGBT older adults in rural southern Appalachia. J Gerontol Soc Work. (2020) 63:768–89. doi: 10.1080/01634372.2020.1774028, 32558626

[33] Alvarez-Hernandez LR Gómez JM. Familismo as structural oppression toward sexual and gender Minoritized Latines: the role of betrayal and double-consciousness in dissociating for the Familia. J Trauma Dissociation. (2026) 27:519–37. doi: 10.1080/15299732.2026.2674556, 42365587

[34] Weaver RH Sano Y Lanigan J Parker L Eddy L Power TG . Perceived changes in social connectedness across the life course: a mixed method approach. Int J Aging Hum Dev. (2023) 96:201–18. doi: 10.1177/00914150221077955, 35137634

[35] Gorczynski P Fasoli F. Loneliness in sexual minority and heterosexual individuals: a comparative meta-analysis. J Gay Lesbian Ment Health. (2022) 26:112–29. doi: 10.1080/19359705.2021.1957742

[36] Repo V Frilund R Kymalainen P. At the end of the rainbow: ageing and place of LGBTQI+ people. Geogr Ann B Hum Geogr. (2026). doi: 10.1080/04353684.2026.2650185

[37] Marhankova JH. Places of invisibility: LGB aging and the (Im)possibilities of coming out to others. J Aging Stud. (2019) 48:9–16. doi: 10.1016/j.jaging.2018.11.002, 30832935

[38] Rosser BRS Shippee T Wright M Aumock C Moone R Talley K . “Going back in the closet”: addressing discrimination against sexual and gender minority residents in long-term services and supports by providing culturally responsive care. J Aging Soc Policy. (2022) 36:875–87. doi: 10.1080/08959420.2023.2226300 PMC10739643 37348486

[39] Cohn-Schwartz E Gooldin S Bachner YG. Gender disparities in the mental health of gay and lesbian adults in later life: the role of internalized homophobia and age discrimination. Sex. Res. Soc. Policy. (2025) 1-11. doi: 10.1007/s13178-025-01175-8, 30311153

[40] Marks AK Ejesi K McCullough MB García Coll C. "Developmental implications of discrimination". In: R. Lerner editor. Handbook of child Psychology and Developmental Science, vol. 3 New York:Wiley (2015). p. 324–65.

[41] Elmer EM van Tilburg T Fokkema T. Age and gender identity in the relationship between minority stress and loneliness: a global sample of sexual and gender minority adults. J Sex Res. (2025) 62:1080–99. doi: 10.1090/00224499.2024.2339511, 38861488

[42] Mahamid F Veronese G Bdier D. Political violence and posttraumatic stress symptoms among Palestinians: the mediating roles of sense of belongingness and loneliness. Int J Psychol. (2023) 58:433–42. doi: 10.1002/ijop.12918, 37208826

[43] Brown AM. Emergent Strategy: Shaping Change, Changing Worlds. Chico, CA: AK Press (2017).

[44] Perone AK Zhou L Reed BG Singh N Kopp-Richardson S Glusker A. A community-engaged scoping review of affordable housing models for LGBTQ+ older adults. The Gerontologist. (2025) 65. doi: 10.1093/geront/gnaf103, 40077852 PMC12082297

[45] Family Eldercare (n.d.). Iris Gardens. (Accessed 17 July 2026). Available online at: https://montrosecenter.org/micro-sites/law-harrington/

[46] Montrose Center (2025). Law Harrington Senior Living Center: There’s no place like home. (Accessed July 17, 2026). Available online at: https://montrosecenter.org/micro-sites/law-harrington/

[47] My Resource Center. Oak Lawn Place: Affirming, Affordable Housing right in Oak Lawn. (Accessed July 17, 2026). Available online at: https://myresourcecenter.org/senior-housing/

[48] Terry E Pharr J Liboro RM Coughenour C Kittle K Waldron J . Identifying and ranking strategies to address housing insecurity and homelessness within the LGBTQIA+ community in southern Nevada: utilization of community-based participatory research and concept mapping. Int J Environ Res Public Health. (2024) 21. doi: 10.3390/ijerph21121540, 39767382 PMC11675821

[49] Rosenwohl-Mack A Smith D Greene M Skultety K Deutsch M Dubbin L . Building H.O.U.S.E (healthy outcomes using a supportive environment): exploring the role of affordable and inclusive housing for LGBTQIA+ older adults. Int J Environ Res Public Health. (2022) 19:1699. doi: 10.3390/ijerph19031699, 35162722 PMC8834975

[50] Services and Advocacy for GLBTQ+ Elders (2026). “SAGECollab.” (Accessed July 17, 2026). Available online at: https://www.sageusa.org/advocacy-partnerships/partnerships/sagecollab/

[51] Perone AK Ingersoll-Dayton B Watkins-Dukhie K. Social isolation loneliness among LGBT older adults: lessons learned from a pilot friendly caller program. Clin Soc Work J. (2020) 48:126–39. doi: 10.1007/s10615-019-00738-8

[52] Caceres BA Streed CG Corliss H Lloyd-Jones D Matthews P Mukherjee M . Assessing and addressing cardiovascular health in lesbian, gay, bisexual, transgender, and queer or questioning (LGBTQ) adults: a scientific statement from the American Heart Association. Circulation. (2020) 142:321–32. doi: 10.1161/CIR.0000000000000914 PMC8212867 33028085

[53] Charise A Pang C Pang C Khalfan KA. What is intergenerational storytelling? Defining the critical issues for aging research in the humanities. J Med Humanit. (2022) 43:615–37. doi: 10.1007/s10912-022-09735-4, 35462580 PMC9759502

[54] Sanjek R. Gray Panthers. Philadelphia, PA: University of Pennsylvania Press (2012).

[55] Perone AK Toman L Reed BG Coldon T Osborne A Cook J. Aging and mentorship in the margins: multigenerational knowledge transfer among LGBTQ+ chosen families. J Gerontol B Psychol Sci Soc Sci. (2025) 80:gbaf027. doi: 10.1093/geronb/gbaf027, 39953965 PMC12067074

[56] Centers for Disease Control and Prevention (CDC). Behavioral Risk Factor Surveillance System Survey Questionnaire. Atlanta, Georgia: U.S. Department of Health and Human Services, Centers for Disease Control and Prevention (2023).

[57] Crystal S Shea DG Reyes AM. Cumulative advantage, cumulative disadvantage, and evolving patterns of late-life inequality. The Gerontologist. (2017) 57:910–20. doi: 10.1093/geront/gnw056, 27030008 PMC5881660

[58] Lee YJ. The impact of the COVID-19 pandemic on vulnerable older adults in the United States. J Gerontol Soc Work. (2020) 63:559–64. doi: 10.1080/01634372.2020.1777240, 32501143

[59] Arenas DJ Thomas A Wang J DeLisser HM. A systematic review and meta-analysis of depression, anxiety, and sleep disorders in US adults with food insecurity. J Gen Intern Med. (2019) 34:2874–82. doi: 10.1007/s11606-019-05202-4, 31385212 PMC6854208

[60] Kahn JR Pearlin LI. Financial strain over the life course and health among older adults. J Health Soc Behav. (2006) 47:17–31. doi: 10.1177/002214650604700102, 16583773

[61] Gibb JK Shokoohi M Salway T Ross LE. Sexual orientation–based disparities in food security among adults in the United States: results from the 2003–2016 NHANES. Am J Clin Nutr. (2021) 114:2006–16. doi: 10.1093/ajcn/nqab290, 34551071

[62] Billies M. Low income LGBTGNC (gender nonconforming) struggles over shelters as public space. ACME: An International Journal for Critical Geographies. (2015) 14:989–1007. doi: 10.14288/acme.v14i4.1125

[63] Romero AP Goldberg SK Vasquez LA. LGBT people and housing affordability, discrimination, and homelessness. UCLA School of Law Williams Institute. (2020) Available online at: https://escholarship.org/uc/item/509184bz

[64] DeFilippis JN. “What about the rest of us?” an overview of LGBT poverty issues and a call to action. J Progress Hum Serv. (2016) 27:143–74. doi: 10.1080/10428232.2016.1198673

[65] Ranahan ME. Planning for the residential needs of lesbian, gay, bisexual, and transgender older adults. J Community Pract. (2017) 25:159–71. doi: 10.1080/10705422.2017.1307299

[66] Kinitz DJ Shahidi FV Kia H MacKinnon K MacEachen E Gesink D . Precarious employment: a neglected issue among lesbian, gay, bisexual, and transgender workers. Sexuality Research and Social Policy. (2025) 22:376–92. doi: 10.1007/s13178-024-00950-3

[67] Emlet CA. Social, economic, and health disparities among LGBT older adults. Generations. (2016) 40:16–22. Available online at: https://www.jstor.org/stable/26556193. 28366981 PMC5373809

[68] United States Census Bureau. Texas population pyramid: population by age and sex. (2024). Available online at: https://data.census.gov/profile/Texas?g=040XX00US48

[69] Hasenbush A Flores A Kastanis A Sears B Gates G. The LGBT divide: a data portrait of LGBT people in the midwestern, mountain & southern states. The Williams Institute. (2014) Available online at: https://escholarship.org/content/qt17m036q5/qt17m036q5_noSplash_76bbb27af26d1e610afb63d07a9c903f.pdf

[70] White Hughto JM Murchison GR Clark K Pachankis JE Reisner SL. Geographic and individual differences in healthcare access for US transgender adults: a multilevel analysis. LGBT health. (2016) 3:424–33. doi: 10.1089/lgbt.2016.0044, 27636030 PMC5165678

[71] Lee JG Wimark T Ortiz KS Sewell KB. Health-related regional and neighborhood correlates of sexual minority concentration: a systematic review. PLoS One. (2018) 13:e0198751. doi: 10.1371/journal.pone.0198751, 29949611 PMC6021065

[72] United Way. 2026. The state of ALICE in Texas: 2026 update on financial hardship. https://www.unitedforalice.org/texas

[73] National Academies of Sciences, Engineering, and Medicine, 2017. Communities in action: pathways to health equity. Consensus study report. https://www.nationalacademies.org/read/24624/chapter/1 28418632

[74] Markides KS Eschbach K. Aging, migration, and mortality: current status of research on the Hispanic paradox. J Gerontol Ser B Psychol Sci Soc Sci. (2005) 60:S68–75. doi: 10.1093/geronb/60.Special_Issue_2.S68, 16251594

[75] Chen J McLaren H Jones M Shams L. The aging experiences of LGBTQ ethnic minority older adults: a systematic review. The Gerontologist. (2022) 62:e162–77. doi: 10.1093/geront/gnaa134, 32941597

[76] Hawkins J Madden K Fletcher A Midgley L Grant A Cox G . Development of a framework for the co-production and prototyping of public health interventions. BMC Public Health. (2017) 17:689. doi: 10.1186/s12889-017-4695-8, 28870192 PMC5583990

[77] Headley MG Plano Clark VL. Multilevel mixed methods research designs: advancing a refined definition. J Mixed Methods Res. (2020) 14:145–63. doi: 10.1177/1558689819844417

[78] Ware JE Sherbourne CD. The MOS 36-ltem short-form health survey (SF-36): I. Conceptual framework and item selection. Med Care. (1992) 30:473–83. https://www.jstor.org/stable/3765916. doi: 10.1097/00005650-199206000-00002, 1593914

[79] Carley S Graff M Konisky DM Memmott T. Behavioral and financial coping strategies among energy-insecure households. Proc Natl Acad Sci. (2022) 119:e2205356119. doi: 10.1073/pnas.2205356119, 36037366 PMC9457251

[80] Pride Center San Antonio. Queer health is the ultimate wealth: San Antonio metro area’s 2024 LGBTQ+ health report. (2024) Available online at: https://pridecentersa.org/wp-content/uploads/2024/11/SCOP-Community-Report-2024-v3-1.pdf

[81] Thorne S. Developing qualitative inquiry. Interpretive description: qualitative research for applied practice (2nd Ed) (2016). doi: 10.4324/9781315545196,

[82] Fritzen-Pedicini C Bleasdale SC Brosseau LM Moritz D Sikka M Stiehl E . Utilizing the focused conversation method in qualitative public health research: a team-based approach. BMC Health Serv Res. (2019) 19:306. doi: 10.1186/s12913-019-4107-0, 31088551 PMC6518626

[83] Rosenfeld D Ramirez-Valles J. Beyond identity and generations: bringing life course theory to studies of older gay men. Front Sociol. (2024) 9:1393607. doi: 10.3389/fsoc.2024.1393607, 38813397 PMC11134570

[84] Clary K Goffnett J King M Hubbard T Kitchen R. “It’s the environment, not me”: experiences shared by transgender and gender diverse adults living in Texas. J Community Psychol. (2023) 51:906–23. doi: 10.1002/jcop.22948, 36206522

[85] Bonomo JA Luo K Ramallo JA. LGBTQ+ cardiovascular health equity: a brief review. Front Cardiovasc Med. (2024) 11:1350603. doi: 10.3389/fcvm.2024.1350603, 38510198 PMC10951381

[86] Hequembourg AL Panagakis C. Maximizing respondent-driven sampling field procedures in the recruitment of sexual minorities for health research. SAGE Open Medicine. (2019) 7:2050312119829983. doi: 10.3389/fsoc.2024.1393607, 30800299 PMC6379799

[87] Texas Department of Housing and Community Affairs. n.d. Texas regions map. Available at: https://www.tdhca.texas.gov/sites/default/files/SFHP-division/htf/docs/09-RefMan-UniformStateSvcRegions.pdf (Accessed July 01, 2026).

